# Cluster analysis: a new approach for identification of underlying risk factors for coronary artery disease in essential hypertensive patients

**DOI:** 10.1038/srep43965

**Published:** 2017-03-07

**Authors:** Qi Guo, Xiaoni Lu, Ya Gao, Jingjing Zhang, Bin Yan, Dan Su, Anqi Song, Xi Zhao, Gang Wang

**Affiliations:** 1 Department of Cardiology, the Second Affiliated Hospital, Xi’an Jiaotong University, Xi’an, China; 2 Shaanxi Engineering Research Center of Medical and Health BIGDATA, School of Management, Xi’an Jiaotong University, Xi’an, China; 3 Department of Emergency Medicine, the Second Affiliated Hospital, Xi’an Jiaotong University, Xi’an, China

## Abstract

Grading of essential hypertension according to blood pressure (BP) level may not adequately reflect clinical heterogeneity of hypertensive patients. This study was carried out to explore clinical phenotypes in essential hypertensive patients using cluster analysis. This study recruited 513 hypertensive patients and evaluated BP variations with ambulatory blood pressure monitoring. Four distinct hypertension groups were identified using cluster analysis: (1) younger male smokers with relatively high BP had the most severe carotid plaque thickness but no coronary artery disease (CAD); (2) older women with relatively low diastolic BP had more diabetes; (3) non-smokers with a low systolic BP level had neither diabetes nor CAD; (4) hypertensive patients with BP reverse dipping were most likely to have CAD but had least severe carotid plaque thickness. In binary logistic analysis, reverse dipping was significantly associated with prevalence of CAD. Cluster analysis was shown to be a feasible approach for investigating the heterogeneity of essential hypertension in clinical studies. BP reverse dipping might be valuable for prediction of CAD in hypertensive patients when compared with carotid plaque thickness. However, large-scale prospective trials with more information of plaque morphology are necessary to further compare the predicative power between BP dipping pattern and carotid plaque.

Hypertension is the most prevalent treatable risk factor, accounting for about 50% of cardiovascular events^1^. Numerous risk factors for coronary artery disease (CAD) have been reported to be associated with hypertension, including overweight, excessive salt intake, and high cholesterol level^2^. Ambulatory blood pressure monitoring (ABPM) can provide much information, including the general level and diurnal rhythm of BP and short- or long-term BP variability, and has potential value in evaluating the prognosis of hypertensive patients^3^. ABPM has contributed to the further understanding of BP fluctuation and demonstrated that hypertensive patients with reverse BP dipping were more prone to have diabetes and metabolic syndrome^4^,^5^.

In addition to BP variability and other clinical risk factors for CAD, carotid plaque load has also been widely used to evaluate the risk and prognosis of cardiovascular disease. Both carotid plaque and intima-media thickness are different measures to assess preclinical arterial pathology, but total plaque area appeared to be a stronger predictor than intima-media thickness for first-ever ischemic stroke^6^,^7^. However, it was not clear whether BP variability, especially reverse dipping, was superior or inferior to carotid plaque in predicting CAD. Therefore, a method for managing multiple variables and their interactions is needed. Cluster analysis, a machine learning approach, has been extensively used in fields such as document classification and image processing^8^. Cluster analysis is a collection of methods for defining subgroups of individuals with high heterogeneity^9^. The groupings are constructed such that the degree of association is strong between members of the same cluster and weak between members of different clusters^10^.

Several reports have suggested that cluster analysis could lead to improved characterization of a disease phenotype^11^,^12^. However, there are few reports of its use to better classify heterogeneous essential hypertension. In this study, we applied cluster analysis to explore possible subgroups within a well-characterized population of hypertensive patients who had circadian BP monitoring and carotid ultrasound examinations. This study would also help to identify potential risk factors for CAD in hypertensive patients.

## Results

### Baseline characteristics of the patient population

**Figure 1:**
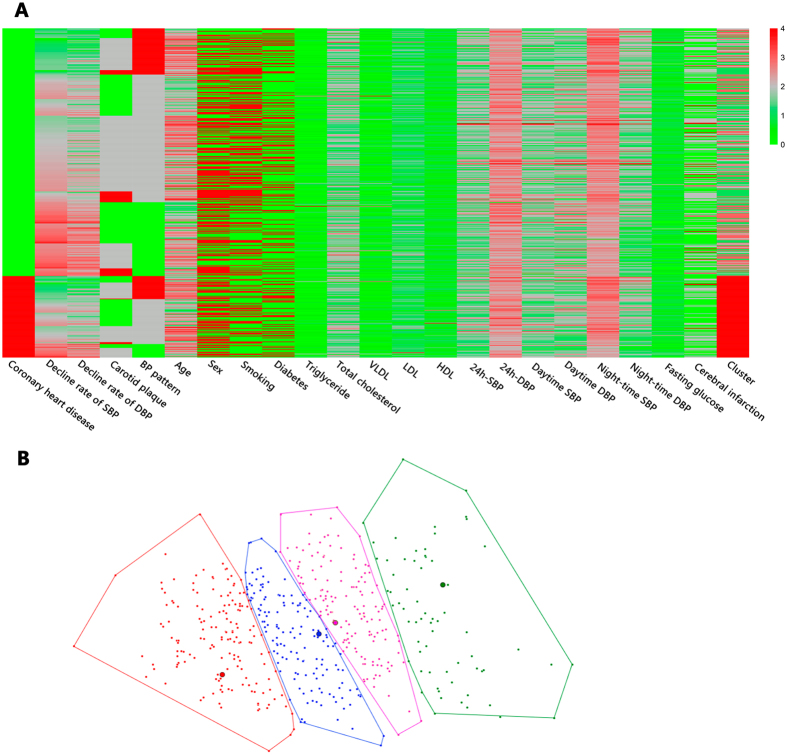
Phenotype heat map (phenomap) and two-dimensional presentation for cluster analysis. (**A**) Within the heat map, rows represent individual study participants while columns represent individual features. (**B**) Cluster results were also shown in two dimensions in order to provide a more intuitive interpretation of clustering. The points with the same colour are divided into the same cluster. VLDL, very low-density lipoprotein; HDL, high-density lipoprotein; LDL, low-density lipoprotein; BP, blood pressure; SBP, systolic blood pressure; DBP, diastolic blood pressure.

A total of 513 patients, including 278 men and 235 women, were enrolled in this study (Fig. 1). The average age of all patients was 61 ± 12 years; 56 patients had incomplete clinical data, including fasting blood glucose and history of cerebral infarction. All included patients had essential hypertension. Comorbidities were common: 143 (27.87%) had diabetes, 211 (41.13%) had cerebral infarction, and 127 (24.75%) had CAD (data not shown).

### Heterogeneity of hypertension

All study participants met common diagnostic criteria for essential hypertension. Nonetheless, a phenotype heat map was created with cluster analysis and demonstrated substantial heterogeneity among all subjects (Fig. 1A). Then we applied principal component analysis to reduce primal data into two dimensions. All patients were eventually divided into four clusters with the same colour using K-means cluster analysis (Fig. 1B).

### Comparison of clinical characteristics among four phenogroups

Cluster analysis identified four groups that were significantly different from each other. Clinical and laboratory characteristics were stratified according to phenogroup (Table 1). Key characteristics of each patient cluster were as follows.

Cluster 1 (n = 172) was the largest, with more than a quarter of all subjects in our study. The patients were smokers and tended to be younger men (average age 59 years) (77.90%). These patients had the lowest level of high density lipoprotein (HDL, 1.18 mmol/L), while the 24-h systolic BP (SBP) (137.31 mmHg), 24-h diastolic BP (DBP) (81.62 mmHg), daytime SBP (139.05 mmHg), daytime DBP (83.33 mmHg), night-time SBP (131.28 mmHg), and night-time DBP (76.69 mmHg) were the highest among the four clusters. These patients were the most likely to have a non-dipper pattern.

Cluster 2 (n = 70) had the lowest number of patients and were primarily older women (average age 64 years) (70.00%). All patients in this cluster were likely to have diabetes mellitus; the levels of fasting glucose (6.72 mmol/L) and total cholesterol (5.03 mmol/L) were the highest. Furthermore, cluster 2 patients had the lowest 24-h DBP (75.34 mmHg), daytime DBP (76.60 mmHg) and night-time DBP (70.47 mmHg). No patients in cluster 1 or 2 had CAD.

Cluster 3 (n = 144) had no smokers or diabetics, and the level of fasting glucose (4.77 mmol/L) was the lowest. Compared with other three clusters, more patients tended to have normal thickness of carotid artery walls (48.61%). Moreover, in this cluster, 24-h SBP (131.84 mmHg), daytime SBP (134.02 mmHg) and night-time SBP (123.80 mmHg) were the lowest. Compared with cluster 1, cluster 3 had the most patients with a dipper pattern (40.97%).

Cluster 4 (n = 127) was the least likely to have severe carotid narrowing (3.15%). Importantly, all of the hypertensive patients in this cluster also had CAD, while patients in the other three clusters had no CAD. The level of total cholesterol (4.47 mmol/L) was the lowest and the level of HDL was the highest (1.32 mmol/L). In addition, cluster 4 had the most patients with a reverse dipper pattern (28.34%) and the rates of decline for both SBP (3.75%) and DBP (6.39%) were the lowest, compared with the other three clusters.

### Association of phenogroups with CAD

**Figure 2:**
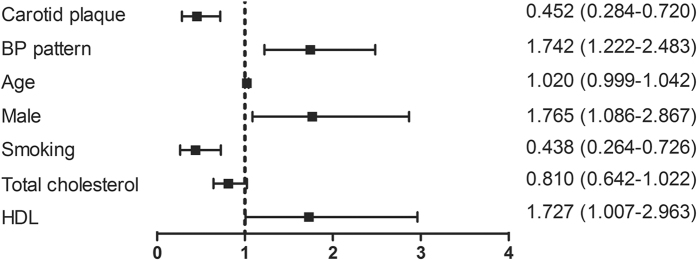
Odds ratio for risk of CAD for the total study population. Risk factors are presented along the left vertical axis and the corresponding odds ratios (95% confidence intervals) are represented along the right vertical axis. BP, blood pressure; HDL, high-density lipoprotein.

To further investigate the risk of CAD in the current patient population, binary logistic regression was carried out after cluster analysis. Multivariate models included variables of age, carotid plaque thickness, sex, smoking, diabetes, CAD, total cholesterol, HDL and BP pattern. For all subjects, carotid plaque (odds ratio [OR] 0.452, 95% confidence interval [CI], 0.284–0.720), BP pattern (OR 1.742, 95% CI, 1.222–2.483), male (OR 1.765, 95% CI, 1.086–2.867), smoking (OR 0.438, 95% CI, 0.264–0.726), and HDL (OR 1.727, 95% CI, 1.007–2.963) were associated with CAD (Fig. 2).

**Figure 3:**
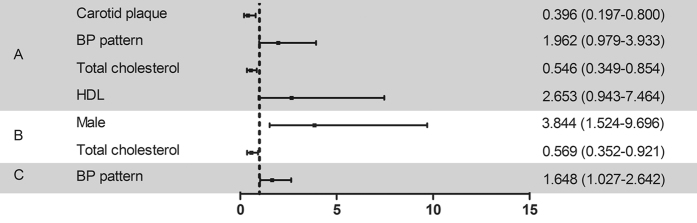
Odds ratio for risk of CAD in a comparison between clusters. (**A**) Comparison between cluster 4 and cluster 1. (**B**) Comparison between cluster 4 and cluster 2. (**C**) Comparison between cluster 4 and cluster 3. Risk factors are presented along the left vertical axis and the corresponding odds ratios (95% confidence intervals) are presented along the right vertical axis. BP, blood pressure; HDL, high-density lipoprotein.

Carotid plaque thickness (OR 0.396, 95% CI, 0.197–0.800) and total cholesterol (OR 0.546, 95% CI, 0.349–0.854) were associated with the risk of CAD in cluster 4 and cluster 1. For cluster 4 and cluster 2, male (OR 3.844, 95% CI, 1.524–9.696) and total cholesterol (OR 0.569, 95% CI, 0.352–0.921) were significantly correlated with CAD. BP pattern (OR 1.648, 95% CI, 1.027–2.642) was the only common risk factor significantly correlated with CAD when comparing cluster 4 with cluster 3 (Fig. 3).

## Discussion

Machine learning approaches are typically subdivided into two categories: supervised and unsupervised learning. Supervised learning seeks to classify or predict specified outputs or outcomes. In contrast, unsupervised learning analyzes the intrinsic structure within data, such as grouping^13^. Among unsupervised learning methods, cluster analysis has been successfully applied in medical research^14^. Using cluster analysis, we were able to take advantage of in-depth phenotyping in our study and found unique patterns of association among phenotypic variables. Although all patients met established criteria for hypertension, the cluster results clearly demonstrate that hypertension is a heterogeneous syndrome. In addition, BP pattern, especially a reverse dipping pattern, showed a great correlation with the risk of CAD in hypertension.

Once the four clusters were identified, the differences between them were striking. Study participants within the four clusters, despite having shared diagnostic features of hypertension, differed markedly on almost every characteristic. In cluster 1, most were younger male smokers, and had the most severe carotid narrowing and relatively high BP; however, none had CAD. Surprisingly, all patients in cluster 4 had CAD, but the least severe carotid narrowing and the most prominent reverse BP dipping pattern. To further investigate the potential significance of reverse dipping pattern, we studied the risk factors for CAD in hypertensive patients, and identified BP pattern as a risk factor. In particular, when comparing cluster 4 with cluster 3, the latter of which was a relatively healthy group, a reverse BP pattern was the only prominent risk factor for CAD. Moreover, carotid narrowing seemed not contribute to the risk of CAD in the results of binary logistic regression. This may be because only carotid plaque thickness were considered, while other parameters of carotid plaque, such as carotid plaque area or three-dimensional plaque characteristics^15^, were not available in this study.

Based on the differences in BP levels between day and night, circadian BP variations were used to divide patients into dippers (mean nocturnal SBP drop of 10 mmHg or more compared with daytime SBP) and non-dippers (all other subjects)^16^. As a specific variant of the non-dipper pattern, the reverse dipper pattern shows a higher night-time SBP compared with daytime values; this pattern was reportedly associated with a higher incidence of cardiovascular events^17^. These findings were consistent with our results for the prognostic value of reverse BP pattern in hypertension. Moreover, in our previous studies, only the reverse dipper BP pattern, rather than the non-dipper pattern, was crucial in the early development of lacunar infarctions^18^.

Assessment of carotid artery plaque with ultrasound examination is a straightforward, inexpensive, and noninvasive method for evaluation of cardiovascular risk^6^. Several studies showed that carotid plaque had significant value in the prediction of ischemic stroke and cardiovascular outcomes^7^,^19^. For examples, carotid plaque area was demonstrated to be a strong risk factor for stroke, myocardial infarction and vascular death, and this predictive value did not be diminished after adjusting for common risk factors^20^. Carotid plaque burden, identified by a new three-dimensional-based ultrasound method, was found to correlate stronger with coronary artery calcium score than IMT^21^. Progression of total plaque volume would be more useful than IMT in the prediction of cardiovascular events or assessment of therapy^22^. Unfortunately, assessment of carotid plaque was limited to carotid plaque thickness rather than total carotid area or volume in our study. Although we found that reverse BP dipping might be valuable in the prediction of CAD in hypertensive patients, the combined use and respective roles of detailed carotid plaque examination and BP dipping status require studies on larger patient populations for verification.

In conclusion, our findings demonstrated the heterogeneity of hypertension with cluster analysis and clearly showed that every subgroup had its own characteristics. Additionally, reverse dipper pattern hypertension was found to contribute to the risk of CAD. However, our study was carried out in a Northern Chinese population with essential hypertension in a single center. Due to the cross-sectional design, prospective clinical observation may also facilitate better understanding of the underlying mechanism.

## Methods

### Study Population

**Figure 4:**
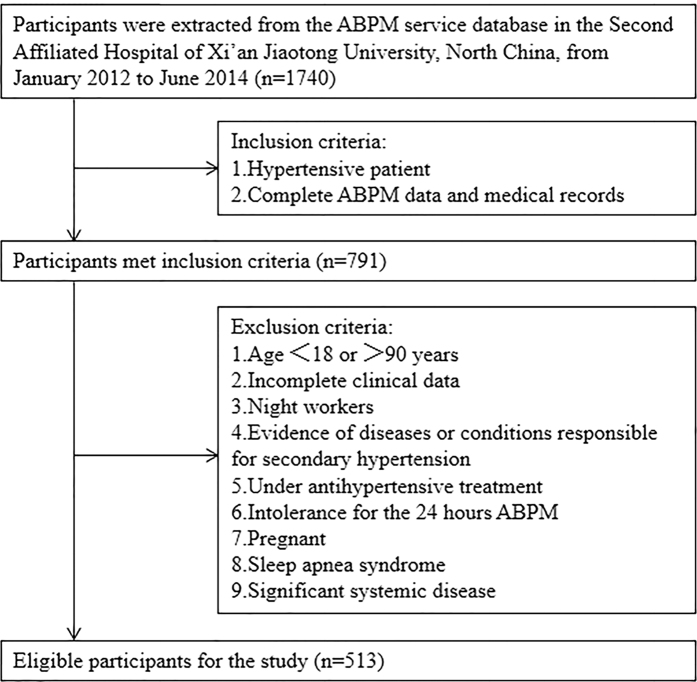
Flow chart showing study population, and inclusion and exclusion criteria. ABPM, ambulatory blood pressure monitoring.

Patients with essential hypertension were recruited continuously from January 2012 to June 2014. Data were extracted from our entire in-patient ABPM service database. Hypertension was diagnosed as systolic BP (SBP) ≥140 mm Hg and/or diastolic BP (DBP) ≥90 mm Hg in casual office recording, or daytime (or awake) SBP ≥ 135 mmHg and/or DBP ≥ 85 mm Hg, or night-time (or asleep) SBP ≥ 120 mm Hg and/or DBP ≥ 70 mm Hg in ABPM^23^. In our study, carotid plaque was defined as a focal thickening that encroaches into the lumen by 0.5 mm or by 50% of the surrounding IMT or where IMT is >1.5 mm^24^. Carotid plaque was examined in diverse segment including proximal common carotid artery (>20 mm proximal to the bulb bifurcation), distal common carotid artery, bulb, internal carotid artery, and external carotid artery on each side and recorded for any thickness >0%. However, information about total plaque area were not obtained in this study. The degree of carotid plaque thickness was divided into normal, 1%–24% narrowing, and ≥25% narrowing. The initial diagnostic approach for CAD includes syndrome, chest radiography, echocardiography and electrocardiogram. After the initial evaluation, stress testing, CT angiography or coronary angiogram was performed for further verification in patients with atypical symptoms according to the guideline^25^. Diabetes mellitus was defined as fasting blood glucose ≥126 mg/dl, casual blood glucose ≥200 mg/dl, or 2-hour blood glucose ≥200 mg/dl during a 75 g oral glucose tolerance test, or previous therapy for diabetes mellitus^26^. Smoking status was ascertained on the basis of self-reported history of cigarette smoking, according to whether smoking consecutive or accumulated more than six months or not^27^. The exclusion criteria included age <18 or >90 years, incomplete clinical data, night workers, diagnosed as secondary hypertension, under antihypertensive treatment, intolerance for the 24 hours ABPM, pregnant, sleep apnea syndrome and significant systemic disease. Eventually, 513 hypertensive patients in total were included in our study. Physical examination and ABPM was performed for all patients (Fig. 4).

### ABPM Assessment

All hypertensive patients were evaluated with 24-hour ABPM using an oscillometric device (Spacelabs 90207; Spacelabs, Redmond, WA). The monitor was installed on the non-dominant arm between 7:00 and 9:00AM and removed 24 hours later. Frequency of recordings was made at every 15 minutes from 7:00AM to 11:00PM (diurnal BP values) and every 30 minutes from 11:00PM to 7:00AM (nocturnal BP values). Strenuous physical activity was discouraged for all patients during the monitoring period. Recordings were accepted only if more than 70% of the raw data was valid. All ABPM recordings were manually edited by 2 individual physicians unaware of the objective and risk factors. Values of SBP < 70 or >250 mm Hg, DBP < 40 or >150 mm Hg, and heart rate < 40 or >150 beats per minute were excluded from the recording. The percent change in nocturnal BP decline was calculated as follows: (mean diurnal BP - mean nocturnal BP)/mean diurnal BP × 100%. According to the BP pattern, patients were divided into three groups: dippers (average SBP decreased 10% to 20% of daytime level during sleep), non-dippers (<10% nocturnal SBP fall) and reverse dippers (SBP nocturnal rise)^28^.

### Cluster analysis

We used the WEKA machine learning toolkit (version 3.6, University of Waikato, Hamilton, New Zealand) for cluster analysis. K-means, one of the widely adopted clustering algorithms, was carried out to find structure in data and divide patients into different groups. Principal component analysis, mapping high-dimension data into low-dimension space, was used to reduce the primal data into two dimensions. As is necessary for clustering analysis, the missing values were globally replaced with mean values. The main steps in the K-means algorithm are as follows: (1) Select initial cluster centres with the number of K. Then, repeat steps 2 and 3 until cluster membership stabilizes. (2) Assign each point to its closest cluster centre. (3) Compute new cluster centres. In step 1, K points are selected randomly as initial cluster centres. In step 2, when we assign each point to its closest cluster centre, we need to compute the distance, typically the Euclidean distance, between points and centres. In step 3, the new cluster centres are computed as the means of all points belonging to each cluster.

### Statistical Analysis

All statistical analyses were performed using the SPSS software package version 18.0 (SPSS Inc., Chicago, IL, USA). Adequacy of all parameters to normal distribution, was tested by using Kolmogorov–Smirnov Test. Parametric tests were applied to data with normal distribution, while non-parametric tests were used to data without normal distribution. Descriptive statistics are presented as percentages for discrete variables and mean ± SD for continuous normally distributed variables. Chi-squared test and analysis of variance (ANOVA) were performed to check the significance of binary and continuous variables respectively between different clusters. Then, binary logistic regression was carried out to investigate the association between CAD and other risk factors. A calculated difference of *P* < 0.05 was considered to be statistically significant.

All of the examinations were carried out for diagnosis of hypertension and assessment of cardiovascular risk in adults in according to guidelines from the European Society of Cardiology^25^. The study protocol was approved by the ethics committee of the Second Affiliated Hospital, Xi’an Jiaotong University, in compliance with the Declaration of Helsinki. All the participants were informed of the nature and purpose of the study and signed written informed consent.

## Additional Information

**How to cite this article:** Guo, Q. *et al*. Cluster analysis: a new approach for identification of underlying risk factors for coronary artery disease in essential hypertensive patients. *Sci. Rep.* **7**, 43965; doi: 10.1038/srep43965 (2017).

**Publisher's note:** Springer Nature remains neutral with regard to jurisdictional claims in published maps and institutional affiliations.

## Figures and Tables

**Table 1:** Clinical and Laboratory Characteristics Stratified by Phenogroup.

Variable	Cluster1 (n = 172)	Cluster2 (n = 70)	Cluster3 (n = 144)	Cluster4 (n = 127)	P
Clinical					
Age, year	59.21 ± 12.31	64.82 ± 12.55	61.35 ± 11.93	62.84 ± 12.68	0.006
Carotid plaque thickness, n (%)					<0.001
normal	56 (32.55)	17 (24.28)	70 (48.61)	58 (45.66)	
1–24% narrowing	90 (52.32)	49 (70.00)	68 (47.22)	65 (51.18)	
≥ 25% narrowing	26 (15.11)	4 (5.71)	6 (4.16)	4 (3.15)	
Male, n (%)	134 (77.90)	21 (30.00)	53 (36.80)	70 (55.11)	<0.001
Smoking, n (%)	172 (100.00)	11 (15.71)	0 (0.00)	39 (30.70)	<0.001
Diabetes mellitus, n (%)	34 (19.76)	70 (100.00)	0 (0.00)	39 (30.70)	<0.001
CAD patients, n (%)	0 (0.00)	0 (0.00)	0 (0.00)	127 (100.00)	<0.001
Cerebral infarction, n (%)	76 (44.18)	21 (30.00)	64 (44.44)	50 (39.37)	0.167
Laboratory data					
Triglycerides, mmol/L	1.89 ± 1.48	1.99 ± 1.13	1.94 ± 1.85	1.85 ± 1.34	0.918
Total cholesterol, mmol/L	4.56 ± 1.00	5.03 ± 1.01	4.62 ± 0.97	4.47 ± 0.97	0.002
VLDL, mmol/L	0.64 ± 0.63	0.74 ± 0.47	0.66 ± 0.56	0.63 ± 0.54	0.592
LDL, mmol/L	2.70 ± 0.86	2.99 ± 0.82	2.71 ± 0.89	2.63 ± 1.00	0.056
HDL, mmol/L	1.18 ± 0.29	1.26 ± 0.29	1.29 ± 0.29	1.32 ± 0.61	0.017
Fasting glucose, mmol/L	5.25 ± 2.37	6.72 ± 2.68	4.77 ± 1.01	5.53 ± 1.81	<0.001
ABPM results					
24 h-SBP, mmHg	137.31 ± 14.19	135.05 ± 12.89	131.84 ± 12.92	133.57 ± 13.96	0.004
24 h-DBP, mmHg	81.62 ± 11.04	75.34 ± 10.18	77.56 ± 9.68	77.73 ± 10.19	<0.001
Daytime SBP, mmHg	139.05 ± 14.36	136.48 ± 13.12	134.02 ± 13.49	136.67 ± 13.78	0.006
Daytime DBP, mmHg	83.33 ± 9.94	76.60 ± 10.26	79.03 ± 9.89	78.48 ± 9.80	<0.001
Night-time SBP, mmHg	131.28 ± 16.47	128.35 ± 20.21	123.80 ± 13.60	129.60 ± 16.29	0.001
Night-time DBP, mmHg	76.69 ± 10.06	70.47 ± 10.71	71.63 ± 9.97	73.25 ± 10.68	<0.001
Circadian BP pattern, n (%)					<0.001
reverse dipper	31 (18.02)	16 (22.85)	25 (17.36)	36 (28.34)	
non-dipper	104 (60.46)	35 (50.00)	60 (41.66)	70 (55.11)	
dipper	37 (21.51)	19 (27.14)	59 (40.97)	21 (16.53)	
Circadian decline rate of SBP, %	5.46 ± 6.74	4.93 ± 7.60	7.34 ± 6.90	3.75 ± 7.01	<0.001
Circadian decline rate of DBP,%	8.07 ± 6.96	7.77 ± 8.63	9.19 ± 8.20	6.39 ± 8.43	0.037

Categorical variables are presented as counts and percentages; continuous variables are presented as mean ± SD. CAD, coronary artery disease; VLDL, very low-density lipoprotein; HDL, high density lipoprotein; ABPM, Ambulatory blood pressure monitoring; LDL, low-density lipoprotein; BP, blood pressure; SBP, systolic blood pressure; DBP, diastolic blood pressure.
